# Ten simple rules for a successful global health roundtable

**DOI:** 10.1371/journal.pcbi.1014916

**Published:** 2026-11-05

**Authors:** Maria Gabriela Uribe Guajardo, Amanda Rush, Michelle Cunich, Kathleen Cravero, Carmen Huckel Schneider

**Affiliations:** 1 The Leeder Centre for Health Policy, Economics and Data, Sydney School of Public Health, University of Sydney, Sydney, Australia; 2 Charles Perkins Centre, Central Clinical School, Faculty of Medicine and Health, The University of Sydney, Sydney, Australia; 3 Cardiovascular Initiative, Faculty of Medicine and Health, The University of Sydney, Sydney, Australia; 4 Sydney Institute for Women, Children and their Families, Sydney Local Health District, Sydney, Australia; 5 NSW Health, Sydney Local Health District, Sydney, Australia; 6 Center for Immigrant, Refugee and Global Health, City University of New York Graduate School of Public Health and Health Policy, New York City, New York, United States of America; Dassault Systemes BIOVIA Ltd, UNITED STATES OF AMERICA

## Abstract

Qualitative approaches play a critical role in global health research by capturing the social, political, and contextual dimensions that shape health outcomes. While interviews and focus groups are widely used, these methods are often time‑limited and project‑specific. Global health roundtables offer a complementary approach, providing a flexible and long-term platform for engaging diverse stakeholders—including researchers, practitioners, policymakers, and community representatives—in collective dialogue, knowledge exchange, and collaboration. When designed well, roundtables can support co‑production of knowledge, address complex system‑level challenges, and generate insights with relevance beyond a single event. Despite their growing use, practical guidance on how to design and run roundtables specifically for global health remains limited. In this article, we present **ten simple rules for organising successful global health roundtables**, drawing on existing literature, practice‑based experience, and examples from different geopolitical and health‑system contexts. The rules address key stages of the roundtable lifecycle, including topic selection, objective setting, inclusive participation, management of power dynamics, optimisation of engagement and commitment, ethical recognition of contributions, and translation of dialogue into meaningful outputs and sustained collaboration. Together, these rules provide an accessible framework to support researchers, practitioners, and policy actors in using roundtables as ethical, inclusive, and impactful tools for global health research, policy development, and practice.

## Introduction

Qualitative research methods are especially valuable in guiding global health analysis [1,2]. They allow researchers to explore the diverse cultural, political, and social experiences, perceptions, and behaviours of participants—insights that are crucial for shaping global health relevance. While stakeholder interviews and focus groups are commonly used to collect such data, these approaches are often short-term and project-specific [3].

Global roundtables, by contrast, offer a more sustainable and integrative platform for engaging a wide range of disciplines and stakeholders, including community representatives [4].

For the purposes of this article, a global health roundtable is defined as a structured and facilitated discussion that brings together researchers, practitioners, policymakers, community representatives, and people with lived experience from diverse geographical and institutional contexts to exchange knowledge, build collaborative relationships, and jointly address complex global health challenges [5,6].

This format is gaining traction as a means of fostering ongoing dialogue, generating insights relevant to global health, and promoting inclusive knowledge exchange (see Table 1). Roundtables encourage collaboration with impact that can extend well beyond a single event [7–9].

**Table 1:** Comparison of roundtable, interview, and focus group methods for global health research.

Method	Key features	Strengths	Weaknesses
**Roundtable**	- Structured discussion among diverse stakeholders - Often used for policy, strategy, or cross-sectoral collaboration beyond a single project - Can include researchers, practitioners, decision-makers, and participants with lived experience - Global scope	- Facilitates multi-perspective dialogue - Useful for identifying systemic challenges and priorities - Promotes consensus and networking	- May lack depth on individual experiences - Risk of dominant voices - Requires skilled facilitation to balance power dynamics
**Focus group**	- Guided discussion among a small group - Often grouped by shared traits (e.g., profession, region, and health condition) - Led by a facilitator - More localised scope	- Reveals collective attitudes and norms - Efficient for gathering multiple viewpoints - Stimulates discussion and idea generation	- May inhibit disclosure of sensitive or personal issues - Risk of groupthink or dominant participants - Requires careful group composition
**Interview**	- One-on-one conversation - Can be structured, semi-structured, or unstructured - Often used for in-depth exploration - Individual scope	- Provides rich, detailed insights - Suitable for sensitive topics and vulnerable populations - Flexible across contexts and cultures	- Time and resource intensive - Limited scalability - Requires skilled interviewers and cultural sensitivity

Serial roundtables further enhance this value, enabling reflection and evolution of discussions over time—even with consistent participants. They help balance diverse voices, treating stakeholders as equal partners in the co-production and translation of knowledge. Roundtables also facilitate the sharing of multiple knowledge domains—experimental, observational, contextual, applied, and experiential—which are all essential when addressing complex global health challenges [10].

Roundtables provide the opportunity to promote both formal and informal conversations, supporting collaborations and networking among experts and across communities, with impact potentially extending beyond a single event [7–9]. They provide valuable opportunities for problem solving. The PLOS Computational Biology Ten Simple Rules collection has previously offered guidance on organising special sessions at scientific conferences [11], hosting inclusive conferences [12], building global networks in computational biology [13], and avoiding the perpetuation of helicopter research in the Global South by Global North researchers [14]. However, an important gap remains as to how to design and run a roundtable specifically focussed on global health. In this article, we present ten rules to support the effective planning, delivery, and utilisation of the diverse insights that global health roundtables can produce.

The ten rules presented were developed through a synthesis of the authors’ collective experience designing, convening, facilitating, and evaluating roundtables across global health research, policy, and practice settings [6,15]. Through repeated implementation of roundtables involving researchers, policymakers, practitioners, community representatives, and people with lived experience, the authors identified recurring challenges and factors associated with successful roundtable design and delivery.

## Rules

A figure outlining the rules for before, during, and after the roundtable is depicted in Fig 1.

**Fig 1:**
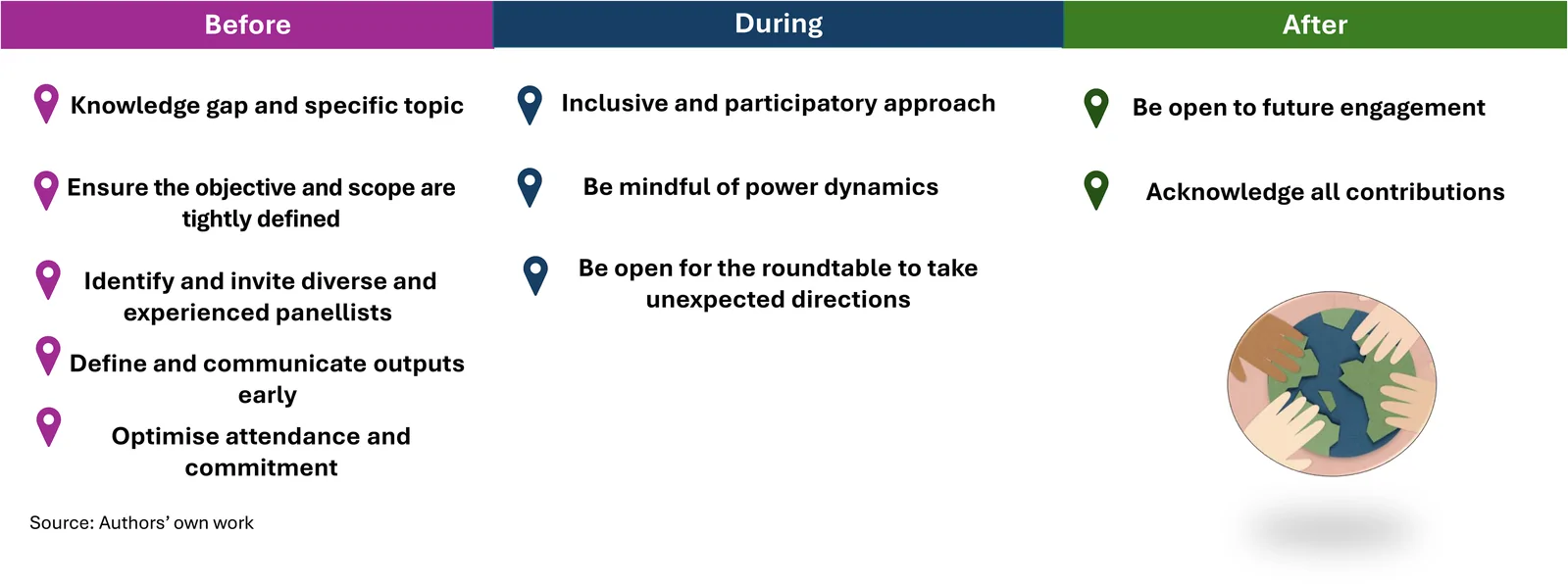
Ten simple rules for a global health roundtabel: before, during and after.

### ① Choose a knowledge gap and specific topic; follow the ‘Ten simple rules for organising a special session at a scientific conference’ by Davide Chicco and Philip E. Bourne

The choice of topic is foundational to the success of any roundtable. They should be neither overly broad nor diffuse; instead, it must be clearly defined and anchored within a specific subfield of the wider scientific domain of the event or gap [9]. A well‑chosen topic reflects current momentum in the literature—emerging, timely, and positioned at the cutting edge of the field. It might be that a series of roundtables is required to address different sub-topics of one larger issue and/or several related issues related to a broader inquiry. The relevant topic should be clearly linked to the roundtable objective and the questions.

Moreover, in global health, focussing the topic using specific expert cohorts, such as academics or professionals [6,16], people with lived experience and community leaders or consumer representatives [17], or a combination of all perspectives [16] could be valuable.

Roundtable topics are well suited to addressing region‑specific health system challenges, with topics commonly organised by world area and geopolitical context, including the Global South, Global North, Europe, North America, and the Western Pacific. This regional tailoring reflects differences in health system structures, governance arrangements, and policy priorities, and political environments which shape the issues most relevant for discussion in each region.

Health roundtables are a flexible way to focus discussions when assessing structures in different parts of the world. In the Global North, they are often used to bring together experts to discuss advanced health systems, new clinical approaches, and digital health governance [18,19]. In Europe and North America, roundtables have supported conversations on vaccination policy, how health data are used, and how to address health inequities [20,21]. In the Western Pacific, particularly Australia, New Zealand, and Japan, roundtables have focussed on chronic disease care and improving health‑system practice [22]. Across Global South and LMIC‑relevant settings, WHO‑led roundtables have increasingly concentrated on topics related to strengthening health systems, improving equity, and developing practical digital health solutions that can be scaled across countries [23].

### ② Ensure the objective, scope, and discussion questions are tightly defined

Once the broad topic has been set, the next step is to clearly define the roundtable’s objective. This needs to happen early, as many later planning decisions depend on it. The objective might be to advance specific knowledge, build networks, support collaboration, or inform future research or policy. Whatever the aim, it should be focussed, relevant, and ideally linked to an emerging area of work or a clearly defined project. In some cases, a series of roundtables may be more appropriate to explore different aspects of a larger issue or related themes under a shared umbrella.

This objective should be shared with panellists well in advance so they understand the purpose of the discussion and can prepare appropriately. A clear objective provides direction, keeps contributions aligned, and helps ensure the discussion generates useful outcomes rather than remaining broad or unfocussed.

With the objective agreed, the next step is to develop discussion questions that directly support it. Questions should be carefully designed to generate clear, practical insights within the time available. Strong questions should be focussed enough to be answered concisely, but open enough to encourage debate and diverse perspectives. Think of them as conversation starters with a purpose.

### ③ Identify and invite diverse and experienced panellists

Before extending invitations, it is important to clearly define the range of expertise required, which will depend on the roundtable topic and objective. A key condition for a ‘wise crowd’ is diversity of expertise, as groups make better decisions when they include a wide range of perspectives and backgrounds [23].

In global health, this means including voices from across the system, with consideration of individuals with lived experience, caregivers, consumer leaders or representatives, frontline workers, clinicians, academics, administrators, policy‑makers, and decision‑makers. Diversity should also be considered within professional groups, as including different specialities, disciplines, and roles (for example, across health professions) adds depth and richness to discussion.

### ④ Define outputs arising from the roundtable early on and communicate them to relevant stakeholders

Roundtables are convened to illuminate complex issues through discussion and debate across sectors. In practice, they can generate a range of academic [6] or nonacademic [8] tangible outputs, including planning/activities, creation of steering committees, memorandums of understanding, guidelines, health reports, policy briefs, peer‑reviewed articles, commentaries, editorials, white papers, and advocacy initiatives. Such outputs provide valuable opportunities for discovery, synthesis, and critical cross‑disciplinary thinking, and can help translate dialogue into broader academic, policy, or practice impact.

To support this process, it is important to clarify at an early stage whether outputs are intended to follow from the roundtable and, if so, to communicate these plans clearly to participants and relevant stakeholders. This includes identifying the nature of the anticipated outputs, assigning responsibility for leading the writing‑up process, and clarifying roles related to coordination, correspondence, authorship, and submission, where applicable.

While roundtable‑generated outputs can represent important sources of global or cross‑sectoral knowledge, they may offer less depth than data derived from methods such as interviews or focus groups, which are more likely to capture individual or localised experiences in detail. Being transparent about both the intended outputs and the methodological strengths and limitations of roundtable discussions helps ensure appropriate interpretation, ethical use of contributions, and alignment between expectations and outcomes.

### ⑤ Optimise attendance and commitment

To build momentum and ensure strong participation, panellists should be encouraged not only to commit to attending the roundtable, but also to actively engage before, during, and after the event. Clear communication about expectations—such as preparation time, active participation in discussion, and potential follow‑up activities—helps signal that the roundtable is a meaningful commitment rather than a passive speaking opportunity.

Engagement can be strengthened by encouraging panellists to involve their peers and networks, for example by sharing the event within professional or community circles, recommending additional contributors, or continuing discussions beyond the session itself. Providing incentives can also reinforce buy‑in. These may include emphasising the public profile or reach of the roundtable, opportunities to shape agendas or outputs, professional recognition, or the potential for collaboration, co‑authorship, or future projects.

When panellists feel invested in the purpose and outcomes of the roundtable, they are more likely to champion the process, contribute thoughtfully, and maintain consistent attendance. This sense of shared ownership helps create energy around the discussion and supports stronger, more sustained engagement over time.

### ⑥ Embrace an inclusive and participatory approach, follow ‘the Ten simple rules for Global North researchers to stop perpetuating helicopter research in the Global South’ by Haelewaters, Hofmann & Romero-Olivares

Health roundtables should be designed to share power, value lived experience, and avoid extractive or ‘helicopter’ research practices, particularly when working with the Global South, LMICs, or disadvantaged and minority groups. Global North researchers should follow guidance such as the Ten Simple Rules to Stop Perpetuating Helicopter Research [14], which emphasises local leadership, fair recognition, and long‑term relationships. Roundtables should avoid colonising practices, including imposing Western‑centric research norms or rigid formats that limit participation. Evidence from participatory migration research shows that even participatory frameworks can reinforce power imbalances if they are imposed rather than shaped by communities themselves [6].

Inclusive roundtables actively create space for nonacademic voices, such as frontline workers and people with lived experience, recognising different ways of contributing, and ensuring discussions lead to meaningful outcomes for participants. An inclusive approach is both ethical and essential for producing relevant, high‑quality, and impactful roundtable outcomes.

### ⑦ Be mindful of power dynamics

In global health roundtables, it is essential to actively recognise and manage power imbalances among panellists. Individuals with lived experience, caregivers, frontline workers, or community representatives may feel less confident or less entitled to speak openly when seated alongside senior clinicians, policymakers, or institutional leaders. Without careful facilitation (e.g., the Chair’s or Convenor’s role), these dynamics can lead to quieter voices being undervalued or overlooked, while more senior or confident participants dominate the discussion.

Panel design and facilitation should therefore aim to minimise hierarchies and promote environments where all voices are valued [24]. This can include balancing seniority across the panel, limiting the number of high‑status or dominant voices, clearly setting expectations about equal contributions (e.g., introducing time limits), and using facilitation techniques that invite diverse participation from all panellists. Being intentional about power dynamics helps ensure that a wider range of perspectives is heard, leading to richer discussions and more meaningful, inclusive outcomes.

### ⑧ Be open for discussions to take unexpected directions

Roundtables are not scripted performances but dynamic, evolving conversations. While thorough preparation is essential, it is equally important to allow space for discussions to develop in unexpected directions. Such flexibility can generate some of the most valuable insights. Roundtables may be used to explore emerging trends [13], examine evidence from lived experience (e.g., consumer views) [15], secondary data, or mixed‑methods research, and to invite panellists to endorse, challenge, or reframe their understandings [14]. In this way, they can function as effective tools for triangulation, bringing diverse perspectives together to strengthen evidence through critical reflection and creative exchange.

Careful planning underpins this adaptability. The use of a clear agenda and a detailed run sheet provides necessary structure while allowing sufficient flexibility to respond to unforeseen circumstances. It is therefore important to plan for panellist absence or last‑minute cancellations, including planning for how discussion may continue with fewer contributors. In such cases, the Moderator or Chair may need to intervene by redistributing questions or actively soliciting input from other attendees in order to maintain continuity and depth of discussion. This, in turn, underscores the importance of selecting panellists who are comfortable participating in public dialogue and capable of engaging thoughtfully and flexibly with evolving discussions.

### ⑨ Be open to future engagement, follow the ‘Ten simple rules on how to develop a stakeholder engagement plan’ by Hollmann et al.

Roundtables can be used strategically as catalysts for longer‑term collaboration rather than as discrete, one‑off events [8]. To support this, organisers should plan from the outset how the roundtable will connect to subsequent activities and forms of engagement. This includes clearly articulating the purpose of the roundtable in relation to future work—such as informing policy development, initiating collaborative research, supporting implementation initiatives, or strengthening practitioner–researcher networks [5].

Practical mechanisms can help translate discussion into sustained connection. These may include collecting consent to share participant contact details, establishing an email list or online workspace, or nominating a small coordination group to maintain momentum after the event.

If resources permit, drafting an adapted stakeholder engagement plan as reported by Hollmann *et al*. 2022 [25] can be a good tool for future and sustained engagement. This might include scheduling stakeholder follow‑up meetings, workshops, or working groups to progress specific ideas or initiatives identified during the discussion.

Roundtables (if aligned with original objectives) can be intentionally positioned as entry points into communities of practice, special interest groups and supporting the exchange of resources, shared learning, and opportunities for joint action. Through deliberate design and follow‑up, they can extend beyond dialogue to enable sustained collaboration and practice‑relevant impact.

### ⑩ Acknowledge contributions from communities, NGOs, academics, and services

Ethical global health roundtables require transparent and meaningful recognition of the time, expertise, and intellectual contributions of communities, NGOs, academics, practitioners, service providers and (more importantly) local and often disadvantaged communities [26]. Such contributions often shape problem framing, interpretation, and the usefulness of outputs for policy and practice, and should be acknowledged to avoid extractive engagement and perpetuation of inequities [26]. Ethical practice includes offering co‑authorship or appropriate contributor recognition where warranted, clarifying expectations early, and providing meaningful acknowledgements otherwise. Recognition should extend beyond publications to include support for dissemination, co‑presentation, and capacity‑building, ensuring roundtables function as equitable partnerships grounded in reciprocity, shared ownership, and ethical knowledge co‑production.

## Conclusion

As global and local health challenges become increasingly complex, interconnected, and shaped by multifaceted social, political, and cultural landscapes, there is a growing need for research methods that enable meaningful dialogue, shared learning, and cross‑sector collaboration. Roundtables offer a distinctive and valuable approach for global health research and practice, providing a structured yet flexible, collaborative and inclusive platform through which multiple forms of knowledge—academic, professional, experiential, and community‑based—can be brought into conversation. When designed well, roundtables can move beyond one‑off discussions to support sustained engagement, co‑production of knowledge, and translation of insights into policy and practice.

However, the effectiveness of global health roundtables should not be taken for granted: successful roundtables require deliberate planning, ethical reflection, and active facilitation. Decisions related to topic selection, objectives, participant diversity, power dynamics, expectations for engagement, and outputs all shape whether a roundtable becomes a meaningful collaborative space or a passive, extractive exercise. Without careful attention to these elements, roundtables risk reproducing existing hierarchies, privileging dominant voices, or delivering limited value to participants and communities.

The ten simple rules presented here are intended as practical guidance to support researchers, practitioners, and policy actors in designing and delivering global health roundtables that are inclusive, purposeful, and impactful. Together, these rules emphasise clarity of intent, respect for diverse forms of expertise, ethical recognition of contributions, and openness to outcomes that extend beyond the event itself. Applied collectively and adaptively, they can help organisers maximise participation, foster trust, and strengthen the relevance and legitimacy of roundtable‑generated insights.

Ultimately, global health roundtables should be understood not merely as events, but as relational processes embedded within broader systems of engagement and knowledge exchange. They can be powerful entry points for collaboration, communities of practice, and longer‑term partnerships that contribute to more equitable, responsive, and impactful global health research and policymaking.

## References

[1] Tembo D, Hickey G, Montenegro C, Chandler D, Nelson E, Porter K, et al. Effective engagement and involvement with community stakeholders in the co-production of global health research. BMJ. 2021;372:n178. doi: 10.1136/bmj.n178 33593805 PMC7879275

[2] Jacobson J, Brooks A. Reflections on “Orchestrating for Impact”: harmonizing across stakeholders to accelerate global health gains. Am J Trop Med Hyg. 2022;106(5_Suppl):67–9. doi: 10.4269/ajtmh.21-1101 35292575 PMC9154649

[3] Busetto L, Wick W, Gumbinger C. How to use and assess qualitative research methods. Neurol Res Pract. 2020;2:14. doi: 10.1186/s42466-020-00059-z 33324920 PMC7650082

[4] World Health Organization. Strategic roundtables: seventy-seventh world health assembly. World Health Organization; 2024. Available from: https://www.who.int/news-room/events/detail/2024/05/28/default-calendar/strategic-roundtables-seventy-seventh-world-health-assembly

[5] Romich J, Fentress T. The policy roundtable model: encouraging scholar–practitioner collaborations to address poverty-related social problems. J Soc Serv Res. 2019;45(1):76–86. doi: 10.1080/01488376.2018.1479343

[6] Uribe Guajardo M, Diab JL, Curry G, Lamarque M, Mazariego J, Briozzo E, et al. Advancing Participatory approaches in global migrant research: a roundtable discussion. J Immigr Minor Health. 2025;28(3):768–74. doi: 10.1007/s10903-025-01798-4 41152661

[7] Davis M. Scientific papers and presentations: navigating scientific communication in today’s world. Group Communications. 2nd ed. Burlington, MA: Academic Press; 2005. pp. 205–14.

[8] Pennel CL, Carpender SK, Quiram BJ. Rural health roundtables: a strategy for collaborative engagement in and between rural communities. Rural Remote Health. 2008;8(4):1054. doi: 10.22605/rrh1054 19025319

[9] McGinnis JM, Aisner D, Olsen L. The learning healthcare system: workshop summary. Washington (DC): National Academies Press; 2007. 21452449

[10] Institute of Medicine. Informing the future: critical issues in health. 3rd ed. Washington, DC: The National Academies Press; 2005.

[11] Chicco D, Bourne PE. Ten simple rules for organizing a special session at a scientific conference. PLoS Comput Biol. 2022;18(8):e1010395. doi: 10.1371/journal.pcbi.1010395 36006874 PMC9409505

[12] Joo R, Sánchez-Tapia A, Mortara S, Bellini Saibene Y, Turner H, Hug Peter D, et al. Ten simple rules to host an inclusive conference. PLoS Comput Biol. 2022;18(7):e1010164. doi: 10.1371/journal.pcbi.1010164 35862309 PMC9302732

[13] Adams JI, Ferebee T, Minto M, Pennerman KK, Chambwe N. Ten simple rules for creating a global network in computational biology. PLoS Comput Biol. 2022;18(10):e1010528. doi: 10.1371/journal.pcbi.1010528 36301818 PMC9612568

[14] Haelewaters D, Hofmann TA, Romero-Olivares AL. Ten simple rules for Global North researchers to stop perpetuating helicopter research in the Global South. PLoS Comput Biol. 2021;17(8):e1009277. doi: 10.1371/journal.pcbi.1009277 34411090 PMC8376010

[15] Uribe Guajardo G, Curry G, Lamarque M, Mazariego J, Briozzo E, Stojanovski K. Saying the quiet part out loud: the perspectives of migrant frontline workers on participatory research in migration. J Glob Health. 2026. 10.1007/s10903-025-01798-4 41152661

[16] Wilkinson D, Teli S, Litchfield C, Madeley A, Kelly B, Impey L, et al. Ethics round table: choice and autonomy in obstetrics. J Med Ethics. 2025;51(9):588–92. doi: 10.1136/jme-2024-110503 39613315 PMC12418576

[17] Jenkinson B, Maxwell J, Bell A, Young A, Smith AC, Christoffersen A, et al. Bringing researchers to the consumer table: the process and outcomes of a consumer roundtable on telehealth. J Telemed Telecare. 2025;31(3):430–6. doi: 10.1177/1357633X231188536 37518937

[18] Yap C, Lee Aiyegbusi O, Alger E, Basch E, Bell J, Bhatnagar V, et al. Advancing patient-centric care: integrating patient reported outcomes for tolerability assessment in early phase clinical trials - insights from an expert virtual roundtable. EClinicalMedicine. 2024;76:102838. doi: 10.1016/j.eclinm.2024.102838 39386161 PMC11462221

[19] Kantrowitz JT, Correll CU, Jain R, Cutler AJ. New developments in the treatment of schizophrenia: an expert roundtable. Int J Neuropsychopharmacol. 2023;26(5):322–30. doi: 10.1093/ijnp/pyad011 36932673 PMC10229849

[20] de Waure C, Gärtner BC, Lopalco PL, Puig-Barbera J, Nguyen-Van-Tam JS. Real world evidence for public health decision-making on vaccination policies: perspectives from an expert roundtable. Expert Rev Vaccines. 2024;23(1):27–38. doi: 10.1080/14760584.2023.2290194 38084895

[21] McLemore M, Robles J, Schubert KG, Whitt-Glover MC. How a public health crisis created an impetus for change: the Robert Wood Johnson Foundation’s National Commission to transform public health data systems: expert roundtable. Health Equity. 2022;6(1):777–86. doi: 10.1089/heq.2022.29014.rtd 36225659 PMC9536330

[22] Thomas D, Lewthwaite H, Gibson P, Majellano E, Clark V, Fricker M. Unlocking asthma remission: key insights from an expert roundtable discussion. Respirology. 2025. 10.1111/resp.70047 PMC12128732 40407301

[23] Avital L. WHO-BMJ roundtable: exploring AI-enabled virtual humans in public health. BMJ Innov. 2025;11(3):165–8. doi: 10.1136/bmjinnov-2025-001386

[24] Scurr T, Ganann R, Sibbald SL, Valaitis R, Kothari A. Evaluating public participation in a deliberative dialogue: a single case study. Int J Health Policy Manag. 2022;11(11):2638–50. doi: 10.34172/ijhpm.2022.6588 35247938 PMC9818103

[25] Hollmann S, Regierer B, Bechis J, Tobin L, D’Elia D. Ten simple rules on how to develop a stakeholder engagement plan. PLoS Comput Biol. 2022;18(10):e1010520. doi: 10.1371/journal.pcbi.1010520 36227852 PMC9560496

[26] Sam-Agudu NA, Abimbola S. Using scientific authorship criteria as a tool for equitable inclusion in global health research. BMJ Glob Health. 2021;6(10):e007632. doi: 10.1136/bmjgh-2021-007632 34649868 PMC8506888

